# Missense and nonsense mutations in melanocortin 1 receptor (*MC1R*) gene of different goat breeds: association with red and black coat colour phenotypes but with unexpected evidences

**DOI:** 10.1186/1471-2156-10-47

**Published:** 2009-08-25

**Authors:** Luca Fontanesi, Francesca Beretti, Valentina Riggio, Stefania Dall'Olio, Elena Gómez González, Raffaella Finocchiaro, Roberta Davoli, Vincenzo Russo, Baldassare Portolano

**Affiliations:** 1 DIPROVAL, Sezione di Allevamenti Zootecnici, University of Bologna, Via F.lli Rosselli 107, 42100 Reggio Emilia, Italy; 2 Dep. S.En.Fi.Mi.Zo., Sezione di Produzioni Animali, University of Palermo, Viale delle Scienze – Parco d'Orleans, 90128 Palermo, Italy; 3 ANAFI – Italian Holstein Association, Via Bergamo 292, 26100 Cremona, Italy

## Abstract

**Background:**

*Agouti *and *Extension *loci control the relative amount of eumelanin and pheomelanin production in melanocytes that, in turn, affects pigmentation of skin and hair. The *Extension *locus encodes the melanocortin 1 receptor (MC1R) whose permanent activation, caused by functional mutations, results in black coat colour, whereas other inactivating mutations cause red coat colour in different mammals.

**Results:**

The whole coding region of the *MC1R *gene was sequenced in goats of six different breeds showing different coat colours (Girgentana, white cream with usually small red spots in the face; Maltese, white with black cheeks and ears; Derivata di Siria, solid red; Murciano-Granadina, solid black or solid brown; Camosciata delle Alpi, brown with black stripes; Saanen, white; F_1 _goats and the parental animals). Five single nucleotide polymorphisms (SNPs) were identified: one nonsense mutation (p.Q225X), three missense mutations (p.A81V, p.F250V, and p.C267W), and one silent mutation. The stop codon at position 225 should cause the production of a shorter MC1R protein whose functionality may be altered. These SNPs were investigated in a larger sample of animals belonging to the six breeds. The Girgentana breed was almost fixed for the p.225X allele. However, there was not complete association between the presence of red spots in the face and the presence of this allele in homozygous condition. The same allele was identified in the Derivata di Siria breed. However, its frequency was only 33%, despite the fact that these animals are completely red. The p.267W allele was present in all Murciano-Granadina black goats, whereas it was never identified in the brown ones. Moreover, the same substitution was present in almost all Maltese goats providing evidence of association between this mutation and black coat colour.

**Conclusion:**

According to the results obtained in the investigated goat breeds, *MC1R *mutations may determine eumelanic and pheomelanic phenotypes. However, they are probably not the only factors. In particular, the surprising not complete association of the nonsense mutation (p.Q225X) with red coat colour raises a few hypotheses on the determination of pheomelanic phenotypes in goats that should be further investigated.

## Background

A large number of coat colour phenotypes have been described in different mammalian species. This diversity is due to the presence, distribution and biochemical activity of the melanocytes in which two types of melanin pigments (eumelanins and pheomelanins, that produce black/brown and red/yellow colours, respectively) are synthesized. *Extension *and *Agouti *are the main loci that affect the relative amount of eumelanin and pheomelanin production in these cells [1]. These loci show epistatic interactions in different mammals. Dominant alleles at the *Extension *locus induce black pigmentation, whereas recessive alleles extend the production of pheomelanins, determining red/yellow/pale pigmentation. Mutations at the *Agouti *locus have, in general, opposite models of action, i.e. dominant alleles determine pheomelanic phenotypes, whereas recessive alleles cause black coat colour with a few exceptions.

The *Extension *locus encodes the melanocortin 1 receptor (MC1R), a seven transmembrane domains protein belonging to the G protein coupled receptors [2] that binds the α melanocyte-stimulating hormone (αMSH) inducing eumelanin synthesis. *Agouti*, instead, encodes the agouti signaling protein (ASIP), a paracrine signalling molecule that affects pigmentation acting as antagonist of MC1R, blocking αMSH-receptor interaction and causing a pigment-type switching from eumelanins to pheomelanins [3,4].

Mutations of the *MC1R *gene affecting coat colour have been described in several mammals, such as mice [2], humans [5], guinea pigs [6], cattle [7-9], pigs [10], horses [11], sheep [12], dogs [13,14], foxes [15], bears [16], felids [17], rabbits [18], and pocket mice [19], in which gain of function mutations produce black/dark coat colour, whereas loss of function mutations cause red/yellow or white coat colour.

In goats, a large number of alleles at the *Agouti *locus, accounting for a broad variability on coat colour, has been predicted by classical crossbreeding studies in several breeds [1,20-24]. From these studies, the *Extension *locus does not seem to play a major role on coat colour variability in goats. The existence of a dominant *E*^*D *^black allele and a recessive *e *red allele has been suggested in few breeds [1,25]. In other goat populations, epistatic effects of *Agouti *alleles might mask and confound the action of the *Extension *locus. On the other hand, the wild type *E*^+ ^allele, the most common form supposed at this locus, should make the phenotypic effects of the different *Agouti *alleles possible, as observed in other species [1]. In Boer goats, Wu et al. [26] suggested that a missense mutation (p.K226E amino acid substitution) in the *MC1R *gene was associated with the presence of the red head phenotype. Thus, it seems that, at least in some goat breeds, the mechanisms of determination of the red coat colour might be similar to those already described in other species, in which mutations in the *MC1R *gene are involved in determining this phenotype.

**Figure 1:**
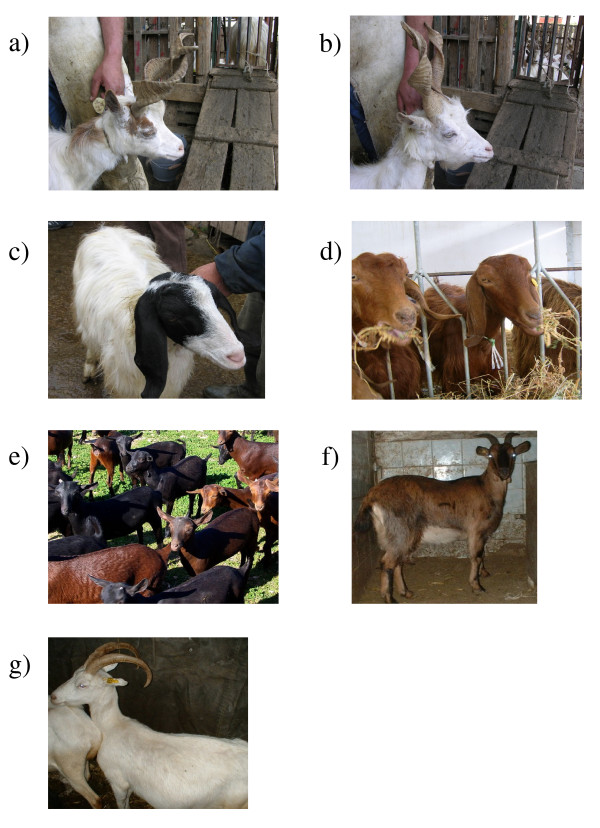
**Investigated goat breeds**. Pictures of (a) Girgentana with red patches, (b) completely white Girgentana, (c) Maltese, (d) Derivata di Siria (Rossa Mediterranea or Mediterranean Red), (e) Murciano-Granadina (with the two colour types), (f) Camosciata delle Alpi, and (g) Saanen goats.

Here, the *MC1R *gene was sequenced and analysed in Girgentana, Maltese, Derivata di Siria (also known as Rossa Mediterranea or Mediterranean Red), Murciano-Granadina, Camosciata delle Alpi, and Saanen goats having different coat colour and patterns (Figure 1), in order to explore the relationship between variations in this gene and coat colour differences among and within breeds. The first three breeds are mainly reared in Sicily (Italy). Girgentana goats, probably of Afghan and Himalayan origin [27], are cream/light-grey with, usually, a few small red spots around eyes and ears, and have long corkscrew horns. This breed is in an endangered status. In ten years, the number of Girgentana goats decreased by 98% [28]. Maltese goats are white with black ears and cheeks, whereas Derivata di Siria animals are solid red. These two breeds have no certain origin. However, it was hypothesised that Maltese originated in Malta, in consequence of crosses between North African and typical Mediterranean breeds, whereas Derivata di Siria was suggested to derive from the Middle East [29]. Murciano-Granadina is one of the most important native Spanish breed originated from the provinces (Murcia and Granada) from which its name comes. The breed is worldwide recognized with the composite name but includes two populations, Murciana and Granadina that might present different characteristics. Traditionally, the Murciana population mainly includes animals with solid brown coat colour (*caoba*), whereas the Granadina population usually includes solid black animals [29,30]. Camosciata delle Alpi is a breed of the Chamois group prevalently distributed in the Alps. Coat colour of these animals is brown with black head, distal portion of the legs, and dorsal stripe [29]. Saanen is a cosmopolitan breed, which originated in Switzerland, with white/cream coat colour probably due to the presence of the dominant *A*^*wt *^(white and tan) *Agouti *allele [20,29].

Our results suggest that mutations we identified in the *MC1R *gene are associated with black and red coat colour, even if not in all breeds, indicating that other genetic factors are important for coat colour determination in the goat.

## Results and discussion

### Identification of mutations

We amplified and sequenced the whole coding region (CDS, 954 bp) and parts of the 5'- and 3'-untranslated regions (38 and 284 bp, respectively) of the *MC1R *gene in 48 goats belonging to the six investigated breeds. The obtained sequences were submitted to the EMBL database under the FM212940 accession number. The CDS encodes a deduced protein of 317 amino acids with 96.8% and 99.4% identity with the bovine and sheep wild type (*E*^+ ^alleles) proteins, respectively.

**Figure 2:**
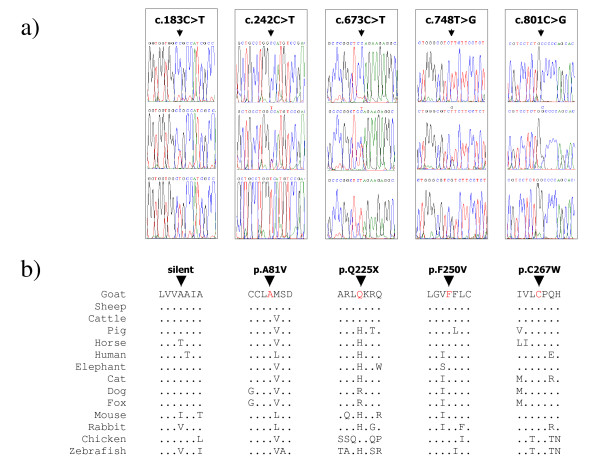
**Identified SNPs and alignment of the MC1R protein regions around the deduced amino acid substitutions**. a) Sequence electropherograms for the five SNPs are reported both for the two homozygous and the heterozygous genotypes. b) Alignment of the goat MC1R protein regions around the corresponding position of the mutated nucleotides with the same protein regions of other species (GenBank accession numbers: sheep, [CAA74298]; cattle, [CAB64818]; pig, [NP_001008690]; horse, [NP_001108006]; human, [NP_002377]; elephant, [ABG37018]; cat, [NP_001009324]; dog, [AAC33737]; fox, [CAA62349]; mouse, [NP_032585]; rabbit, [CAJ57383]; chicken, [BAD91484]; zebrafish, [AAO24742]). The amino acid substitutions for the three missense mutations and the insertion of a stop codon for the nonsense mutation are reported. Dots indicate the same amino acid of the goat protein.

Analysing and comparing the obtained sequence electropherograms, we identified five single nucleotide polymorphisms (SNPs) in the CDS (Figure 2). The most interesting mutation was a c.673C>T substitution that inserts a stop codon at position 225 of the deduced amino acid sequence (p.Q225X). With this mutation, the deduced protein lacks the 93 C-terminal amino acids (including part of the third intracellular loop, the last two transmembrane domains, the last intercellular loop, and the intracellular tail) and for this reason the receptor may not be functional. In other species, insertion of premature stop codons, frame shift or deletions affecting important portions of the MC1R protein are suggested to inactivate the receptor function causing a continuous production of pheomelanin that, in turn, produces red coat colour phenotypes [2,7,8,13,14,18].

Three other missense mutations were identified: 1) c.242C>T, causing a p.A81V substitution; 2) c.748T>G, determining the p.F250V amino acid change; 3) c.801C>G, causing the p.C267W change of amino acid residue. A silent mutation (c.183C>T) was identified at codon 61. One of these SNPs (c.748T>G) was also identified by Wu et al. [26] in a Boer goat. However, the p.K226E MC1R substitution, which was suggested to be associated with red head in Boer goats [26], was not identified in the analysed goats.

**Figure 3:**
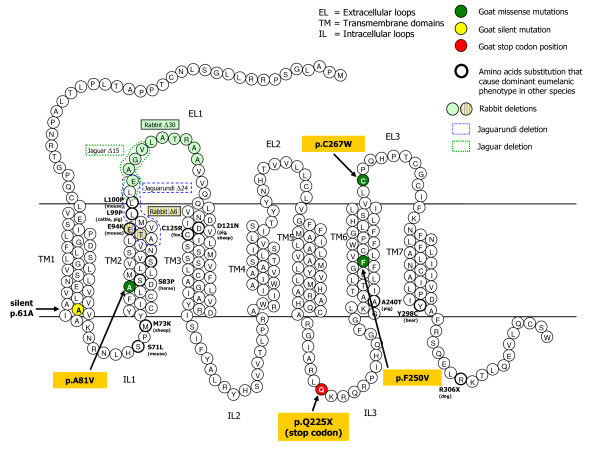
**2D structure of the deduced goat MC1R amino acid sequence with the identified amino acid changes indicated**. Mutations affecting coat colour in other mammals are reported (modified from Majerus and Mundy [52])

Alignments of the deduced goat protein regions around the polymorphic sites with the corresponding MC1R amino acid positions available in other species are reported in Figure 2. Figure 3 reports their positions in the 2D protein structure together with amino acid changes associated with different coat or feather colours in several mammalian or avian species. All three amino acid substitutions are in highly conserved positions across species. Estimation of the likelihood of these non-synonymous (amino-acid changing) coding SNPs to cause a putative functional impact on the protein was evaluated using the cSNP analysis tool of PANTHER (Protein ANalysis THrough Evolutionary Relationships) classification system [31,32], which calculates the subPSEC (substitution position-specific evolutionary conservation) and probability (P_deleterious_) scores based on an alignment of evolutionarily related proteins [31,33] (see Methods section). PANTHER analysis indicated that all these amino acid substitutions may have functional impacts. The p.A81V residue change is located in the second transmembrane-domain, where many mutations affect MC1R activity in several other species. The subPSEC and P_deleterious _scores for this substitution were -6.96288 and 0.98135, respectively. The p.F250V substitution, located in the sixth transmembrane domain, had subPSEC = -5.99812 and P_deleterious _= 0.95249. The p.C267W amino acid variation interested a position in the third extracellular loop, that is one of the most conserved domains of this receptor (Figure 2), resulting in a subPSEC of -5.19272 with P_deleterious _of 0.89959.

### Analysis of the identified mutations in goat breeds with different coat colours

**Figure 4:**
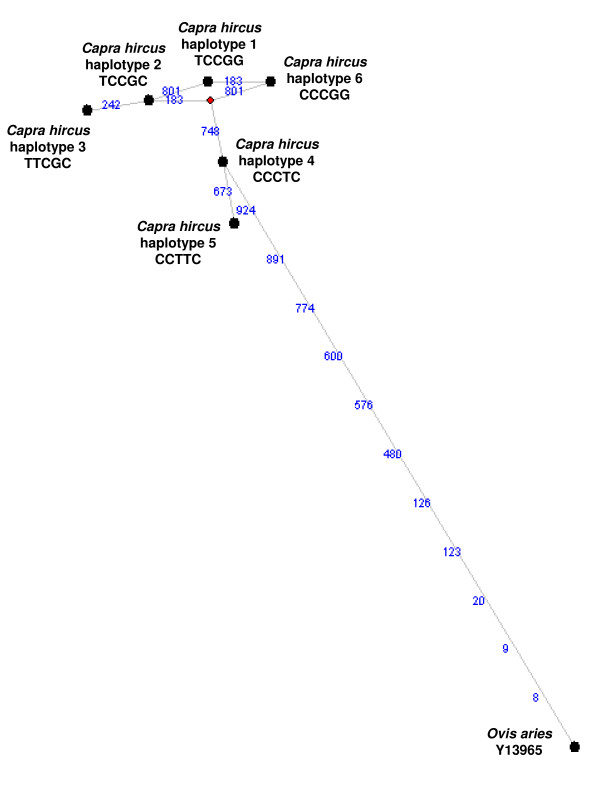
**Median-joining network showing relationships among the five goat *MC1R *haplotypes**. To root the network, the ovine *MC1R *sequence was included. The black circles indicate the goat haplotypes or the ovine sequence. A red square indicates the median vector. Haplotypes are indicated following the SNP position in the *MC1R *gene: c.183C>T; c.242C>T; c.673C>T; c.748T>G; c.801C>G. Branch length is proportional to the number of mutations. Each line is annotated (in blue) with its corresponding mutational change.

To evaluate if the identified mutations were associated with coat colours in the investigated breeds, five PCR-RFLP tests (Additional files 1 and 2) were set up to analyse these SNPs in a larger number of animals (collected from different farms) for a total of 271 goats for which coat colour records were available. Additional 51 Girgentana goats were genotyped. However, they were not considered in the coat colour-genotype association analysis because we did not have any photographic documentation for these animals. Table 1 reports allele and genotype frequencies for the five polymorphic sites and Table 2 reports haplotype frequencies among the investigated breeds. Six haplotypes were identified and a median-joining network, showing their relationships, is reported in Figure 4.

**Table 1:** Allele and genotype frequencies of the identified *MC1R *SNPs in the six investigated goat breeds.

SNP	Breed (no. of animals)^1,2,3,4^	Mutated allele frequency^5^	Genotype frequency (no. of animals)		
p.A61 (c.183C>T)	Girgentana (102)	<0.01	CC = 0.99 (101)	CT = 0.01 (1)	TT = 0.00 (0)
	Maltese (50)	0.94	CC = 0.00 (0)	CT = 0.12 (6)	TT = 0.88 (44)
	Derivata di Siria (39)	0.51	CC = 0.23 (9)	CT = 0.51 (20)	TT = 0.26 (10)
	Murciano-Granadina (28)	1.00	CC = 0.00 (0)	CT = 0.00 (0)	TT = 1.00 (28)
	Camosciata delle Alpi (29)	0.03	CC = 0.93 (27)	CT = 0.07 (2)	TT = 0.00 (0)
	Saanen (18)	0.06	CC = 0.89 (16)	CT = 0.11 (2)	TT = 0.00 (0)
p.A81V (c.242C>T)	Girgentana (102)	<0.01	CC = 0.99 (101)	CT = 0.01 (1)	TT = 0.00 (0)
	Maltese (50)	0.11	CC = 0.78 (39)	CT = 0.22 (11)	TT = 0.00 (0)
	Derivata di Siria (39)	0.50	CC = 0.23 (9)	CT = 0.54 (21)	TT = 0.23 (9)
	Murciano-Granadina (28)	0.00	CC = 1.00 (28)	CT = 0.00 (0)	TT = 0.00 (0)
	Camosciata delle Alpi (29)	0.00	CC = 1.00 (29)	CT = 0.00 (0)	TT = 0.00 (0)
	Saanen (18)	0.00	CC = 1.00 (18)	CT = 0.00 (0)	TT = 0.00 (0)
p.Q225X (c.673C>T)	Girgentana (102)	0.94	CC = 0.01 (1)	CT = 0.11 (11)	TT = 0.88 (90)
	Maltese (50)	0.01	CC = 0.98 (49)	CT = 0.02 (1)	TT = 0.00 (0)
	Derivata di Siria (39)	0.33	CC = 0.49 (19)	CT = 0.36 (14)	TT = 0.15 (6)
	Murciano-Granadina (28)	0.00	CC = 1.00 (28)	CT = 0.00 (0)	TT = 0.00 (0)
	Camosciata delle Alpi (29)	0.00	CC = 1.00 (29)	CT = 0.00 (0)	TT = 0.00 (0)
	Saanen (18)	0.00	CC = 1.00 (18)	CT = 0.00 (0)	TT = 0.00 (0)
p.F250V (c.748T>G)	Girgentana (102)	<0.01	TT = 0.99 (101)	TG = 0.01 (1)	GG = 0.00 (0)
	Maltese (50)	0.97	TT = 0.00 (0)	TG = 0.06 (3)	GG = 0.94 (47)
	Derivata di Siria (39)	0.51	TT = 0.23 (9)	TG = 0.51 (20)	GG = 0.26 (10)
	Murciano-Granadina (28)	1.00	TT = 0.00 (0)	TG = 0.00 (0)	GG = 1.00 (28)
	Camosciata delle Alpi (29)	0.03	TT = 0.93 (27)	TG = 0.07 (2)	GG = 0.00 (0)
	Saanen (18)	0.06	TT = 0.89 (16)	TG = 0.11 (2)	GG = 0.00 (0)
p.C267W (c.801C>G)	Girgentana (102)	0.00	CC = 1.00 (102)	CG = 0.00 (0)	GG = 0.00 (0)
	Maltese (50)	0.76	CC = 0.08 (4)	CG = 0.32 (16)	GG = 0.60 (30)
	Derivata di Siria (39)	0.01	CC = 0.97 (38)	CG = 0.03 (1)	GG = 0.00 (0)
	Murciano-Granadina *Caoba *(15)	0.00	CC = 1.00 (15)	CG = 0.00 (0)	GG = 0.00 (0)
	Murciano-Granadina Black (13)	0.58	CC = 0.00 (0)	CG = 0.85 (11)	GG = 0.15 (2)
	Camosciata delle Alpi (29)	0.00	CC = 1.00 (29)	CG = 0.00 (0)	GG = 0.00 (0)
	Saanen (18)	0.03	CC = 0.94 (17)	CG = 0.06 (1)	GG = 0.00 (0)

^1^Coat colour of these breeds is: Girgentana, white/cream with small red spots in the face (93 animals in our sample), or with small black spots instead of red (1 in our sample), or completely white (8 in our sample); Maltese, white with black checks and ears; Derivata di Siria, solid red; Murciano-Granadina, solid black or solid brown (*caoba*); Camosciata delle Alpi, brown with black stripes; Saanen, white (see Figure 1).

^2^At the c.673C>T site, for the Girgentana breed, considering only the animals with coat colour records (n. = 102) and within this group the three phenotypic types (presence of red spots in the face; presence of black spots in the face; completely white/cream animals), the number of goats for each genotype was: Girgentana with red spots (n. = 93), c.673CT = 3, c.673TT = 90; Girgentana with black spots (n. = 1), c.673CT = 1; Girgentana completely white/cream (n. = 8), c.673CC = 1, c.673CT = 6, c.673TT = 1.

^3^For the Girgentana breed, including the 51 goats for which coat colour records were not available (a total of 153 goats), the number of animals for each genotype at the five polymorphic sites was: c.183CC = 150, c.183CT = 3; c.242CC = 150, c.242CT = 3; c.673CC = 1, c.673CT = 14, c.673TT = 138; c.748TT = 150, c.748TG = 3; c.801CC = 153.

^4^Allele and genotype frequencies for the p.C267W in the Murciano-Granadina breed were separated for black and *caoba *animals.

^5^The mutated alleles were considered to be c.183T, c.242T, c.673T, c.748G, and c.801G.

**Table 2:** Haplotype frequencies at the goat *MC1*R locus

Breed (no. of animals)	Coat colour^1^	**Haplotype frequency**^2^					
		
		1 (TCCGG)	2 (TCCGC)	3 (TTCGC)	4 (CCCTC)	5 (CCTTC)	**6 (CCCGG)**^3^
Girgentana (102)	Usually white with red spots	0.00	0.00	0.01	0.05	0.94	0.00
Maltese (50)	Black	0.73	0.10	0.11	0.02	0.01	0.03
Derivata di Siria (39)	Red	0.01	0.00	0.50	0.15	0.33	0.00
Murciano-Granadina (15)	*Caoba *(brown)	0.00	1.00	0.00	0.00	0.00	0.00
Murciano-Granadina black (13)	Black	0.58	0.42	0.00	0.00	0.00	0.00
Camosciata delle Alpi (29)	Brown	0.00	0.03	0.00	0.97	0.00	0.00
Saanen (18)	White	0.03	0.03	0.00	0.94	0.00	0.00

^1^The complete coat colour description of these breeds is: Girgentana, white/cream with small red spots in the face (93 animals in our sample), or with small black spots instead of red (1 in our sample), or completely white (8 in our sample); Maltese, white with black checks and ears; Derivata di Siria, solid red; Murciano-Granadina, solid black or solid brown (*caoba*); Camosciata delle Alpi, brown with black stripes; Saanen, white (see Figure 1).

^2^Haplotypes are indicated following the SNP position in the *MC1R *gene: c.183C>T; c.242C>T; c.673C>T; c.748T>G; c.801C>G.

^3^Haplotype 6 was observed only in 3 Maltese goats of the Sardinian flock, also carrying haplotype 1.

The nonsense mutation (c.673T; p.225X), present in haplotype 5, was homozygous in 88% and 15% of Girgentana and Derivata di Siria goats, respectively. Among the analysed breeds, Girgentana and Derivata di Siria are the only ones with red colour (red spots around the eyes and ears in Girgentana; Derivata di Siria is solid red; Figure 1). In Girgentana, considering only the animals for which photographic documentation was available (no. = 102), 9 goats did not have the red spot phenotype (completely white, no. = 8; with black spots, no. = 1). Association between the nonsense mutation in homozygous condition and the presence of red spots in Girgentana goats was highly significant (Fisher exact test, *P *= 4.8e-9), even if not complete. Out of the 9 goats without pheomelanic phenotype, 1 white goat was homozygous for the nonsense mutation, 1 white goat was homozygous for the alternative allele (p.225Q), whereas the others were heterozygous (6 white goats and 1 with black spots). In addition, of the 93 Girgentana goats with red spots, 3 were heterozygous and 90 were homozygous for the nonsense mutation (Table 1). In the goats carrying the p.225Q allele, the mechanism that determines white coat colour seems to follow the classical rule of epistatic effects with the *Agouti *locus (or other loci), i.e., the possibility to express the *Agouti *alleles can be obtained only when at least a copy of a putative wild type allele at the *Extension *locus is present. The white phenotype in these goats may be due to the presence of the *A*^*wt *^allele at the *Agouti *locus [34] as described in other goat breeds [1,20,23-25] and in sheep [1,35], even if the biochemical mechanism is not clear yet. However, one completely white animal was homozygous for the nonsense *MC1R *mutation. This might be due to epistatic effects of other loci, including the *Agouti *locus in which different alleles, with putative diverse effects, are present [34]. This incomplete association between the p.225X *MC1R *allele and the pheomelanic phenotype could be due to the fact that the nonsense mutation is not the only factor or the determining factor affecting red coat colour in this breed. The analysis of additional 51 Girgentana goats for which coat colour records were not available, confirmed that the p.225X *MC1R *allele is the most frequent allele in this breed but it is not fixed (Table 1). Thus, the high frequency of the p.225X *MC1R *allele in the Girgentana breed could be due to genetic drift after the bottleneck that this breed experienced a few years ago [28].

An unexpected result that, to some extent, seems to support the latter hypothesis was obtained for the Derivata di Siria breed for which only 15% of the sampled goats were homozygous for the nonsense allele (included only in haplotype 5). According to its phenotype, we might have expected the fixation of the nonsense allele that should cause the production of a non-functional transmembrane receptor, as reported in several other species for similar disrupting mutations [2,7,8,13,14,18]. The *MC1R *haplotype relationships (Figure 4), together with the haplotype distribution in the Derivata di Siria breed, compared to the haplotype frequency in the other breeds (Table 2), might exclude the possible role of other not identified regulatory *MC1R *mutations in red coat colour determination. As a matter of fact, other not identified *MC1R *mutations might have occurred in haplotype 3, haplotype 4, and haplotype 1 (having frequency 50%, 15%, and 1%, respectively) of the Derivata di Siria breed, but not in the same haplotypes observed in other breeds with different coat colour (Table 2). Additional sequencing in the promoter or other regulatory regions could be carried out to confirm this hypothesis.

Two other hypotheses could be considered for the presence of uniform red coat colour in Derivata di Siria, despite its heterogeneity at the *MC1R *gene. One possibility could be that different *MC1R *haplotypes, not related to haplotype 5 (Figure 4), can give the same red coat colour. This would assume dominance or incomplete penetrance of some "red alleles" at the *Extension *locus, for which there is the need to identify a biological explanation. If we would consider this hypothesis, one potential candidate for this effect might be haplotype 3 (50% in Derivata di Siria; Table 2), containing the p.81V substitution for which *in silico *prediction indicated a putative functional role (as reported above). It could be also possible that incomplete penetrance of haplotypes 3 and 5 when in combination with haplotype 4 (the putative wild type haplotype as discussed below; Figure 4) might contribute to the reddish phenotype in these goats. However, we did not note evident differences of intensity of the red colour among animals carrying different haplotype combinations. A second alternative hypothesis could be that other genes are involved in determining red coat colour. In fox, a non-epistatic interaction of *Agouti *and *Extension *was shown to cause red coat colour with the presence of different *MC1R *alleles [15]. However, no mutation in the *Agouti *(*ASIP*) gene seems to be associated with red coat colour in the Derivata di Siria breed [34]. Therefore, other gene(s) involved in (an) alternative mechanism(s) or biochemical pathway(s) that exclude(s) MC1R as the only determinant of the shift between eumelanin and pheomelanin synthesis should be considered. In this goat model, if we reasonable assume that only the truncated MC1R is not functional, we can speculate that an upstream or downstream factor from this transmembrane receptor, independently from its functional or non-functional status, might be the causative or co-causative activator of the pheomelanin synthesis. Recently, a β-defensin protein has been shown to be a ligand of MC1R and competitor of the ASIP protein. Mutations in this gene cause black or brindle coat colour in dog [36]. This might be a possible upstream factor in the goat model even if we should expect an opposite effect in goat respect to what was observed in dog (pheomelanin instead of eumelanin production). Another possible upstream genetic factor could involve the proopiomelanocortin (*POMC*) gene. Sequential cleavage of its coded precursor protein produces the αMSH that binds MC1R and competes with ASIP in regulating, in turn, melanin synthesis. Mutations in the *POMC *gene have been shown to cause red hair pigmentation in humans, together with severe obesity and adrenal insufficiency [37], two defects that so far have not been described in Derivata di Siria goats. At the post-receptor level, pheomelanic signals could be provided either by directly or indirectly controlling intracellular cAMP levels or protein kinase A activity, that are intracellular second-messenger pathways critical for melanogenesis [38].

**Figure 5:**
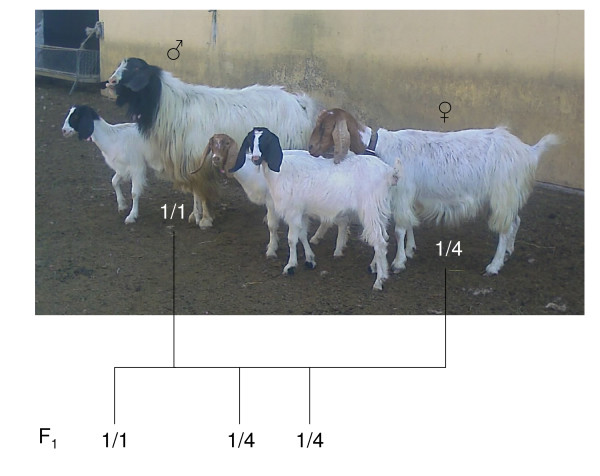
**Segregation of the red coat colour in a goat family and indication of the *MC1R *haplotypes**. A Maltese buck homozygous for haplotype 1 was crossed with a Maltese-like goat (having red head instead of black cheeks and ears) carrying haplotypes 1 and 4. The haplotype of the F_1 _animals is indicated in the correspondence of the goats reported in the picture.

To further investigate this issue, we were able to follow coat colour segregation together with *MC1R *haplotypes in F_1 _animals (2 black and one red) obtained crossing a Maltese buck (black) with a Maltese-like red goat (with red head instead of black cheeks and ears; Figure 5). The parental animals were from a Sardinian flock that was probably admixed in the past with Derivata di Siria blood, as also suggested by the identification of one animal of this flock (with black cheeks and ears) that carried haplotype 5 in combination with haplotype 1 (the only animal with haplotype 5 that was not of Girgentana or Derivata di Siria breed; Tables 1 and 2). The Maltese buck was homozygous for haplotype 1, whereas the red goat carried haplotypes 1 and 4. One F_1 _animal (black) was 1/1 and two were 1/4 (one black and one red; Figure 5). Therefore, in this cross there was no association between coat colour and *MC1R *haplotype combinations, suggesting that other genes might be involved in determining the pheomelanic phenotype, as discussed above.

Other interesting results were obtained for the Murciano-Granadina breed. All black Murciano-Granadina goats were either homozygous or heterozygous for the mutated allele of the c.801C>G SNP, which causes a cysteine to tryptophan change (p.C267W) in a well conserved position (Figure 2), whereas all *caoba *goats of this breed carried only the alternative allele (c.801C). Association between this amino acid substitution and black coat colour was highly significant (Fisher exact test, *P *= 2.7e-8). The putative role of the p.C267W polymorphic site in affecting coat colour is in agreement with the presence of two coat colour populations in the Spanish breed and the Mendelian dominance of the black colour over the *caoba *colour in Murciano-Granadina goats [39]. As reported above, PANTHER analysis indicated that the p.C267W substitution might have a functional role. Cysteine at position 267 should be involved in formation of a disulfide bond with cysteine at position 275 and was indicated to be necessary for the MC1R normal folding and insertion into the membrane [40]. An *in vitro *site-directed mutagenesis experiment, which substituted cysteine with glycine in the human MC1R protein at position 267, resulted in a complete lack of αMSH ligand binding, whereas substitution with serine maintained some binding [40,41]. It is not known if the substitution with tryptophan (this natural goat mutation) might have any effect on ligand affinity. Pharmacological studies are needed to clarify this issue. However, according to the association (at least in the Murciano-Granadina breed) with black coat colour, i.e. eumelanin production, it seems that this substitution might conserve or even strengthen αMSH affinity increasing intracellular cAMP level. Interestingly, a mutation of the cysteine at position 271 in MC4R (corresponding to the p.267C residue in MC1R), was associated with obesity in human [42] supporting again a functional role of this amino acid residue.

The p.C267W mutation, however, seems not to be the only eumelanogenic factor in goats. This assumption derives from the analysis of the Maltese breed (characterized by having black ears and cheeks) and from the F_1 _goats obtained crossing a Maltese buck with a red goat as reported above (Figure 5). Of the 50 analysed goats of this breed, 4 (8%) did not carry the p.267W allele (included in haplotype 1). Two of these goats were heterozygous at the c.242C>T SNP (carriers of haplotypes 2 and 3) and the other two were the only ones heterozygous at the c.748T>G site (carriers of haplotypes 3 and 4) (Tables 1 and 2), even if they had the typical black Maltese pattern (Figure 1). In addition, the F_1 _goats with the same haplotype combination (1/4) had different coat colour (one black and one red; Figure 5).

Moreover, the c.801C>G SNP (p.C267W substitution) was observed in heterozygous condition i) in only one Derivata di Siria goat, further supporting the hypothesis that in this breed *MC1R *gene mutations are not completely associated with coat colour, ii) in one Saanen goat, for which the *Agouti *locus might be the most important genetic factor affecting coat colour [34], and iii) in two Camosciata delle Alpi goats, for which other genes might influence their characteristic phenotype.

Camosciata delle Alpi was almost fixed for haplotype 4 which also showed a high frequency in the Saanen breed. Haplotype 4 might represent a wild-type sequence of the *MC1R *gene (*E*^+ ^allele) that makes the phenotypic effect of the *Agouti *locus possible. As a matter of fact, the coat colour of the Camosciata delle Alpi might be caused by the *a*^*t *^(black and tan) or *A*^*b *^(badgerface) *Agouti *alleles [1,21,23] for which an *E*^+ ^allele would be needed. In the Saanen breed, the expression of the dominant *A*^*wt *^allele [1,20,24,25], similarly, would need a wild-type allele at the *Extension *locus, as discussed above. The supposed wild-type nature of haplotype 4 can also be supported by the median-joining network (Figure 4) in which this haplotype was the closest one to the ovine sequence, used to orient the tree.

Summarizing, as discussed above for the pheomelanic phenotype, also for the eumelanic phenotype other genes, starting from *ASIP *[34], *TYRP *family [43], and, eventually the recently described β-defensin gene [36], may play additional complementary roles to obtain the black colour in goats. Support for this hypothesis comes from classical genetic studies that identified a few other loci with alleles affecting black coat colour in goats [1,21,23,44]. In the Camosciata delle Alpi and Saanen breeds that are almost fixed for a putative wild-type *MC1R *allele, other genes, such as *ASIP *[1,20,21,34], seem to affect their coat colour.

## Conclusion

According to the results obtained in the investigated goat breeds that present different coat colours, *MC1R *mutations may determine eumelanic and pheomelanic phenotypes. However, it seems that the identified *MC1R *alleles are not the only factors, and other upstream or downstream processes might be considered. In particular, the surprising incomplete association of the nonsense mutation (c.673C>T; p.Q225X) with red coat colour phenotype raises two hypotheses that warrant further investigation: i) the presence of a second "red allele" at the *Extension *locus (possibly haplotype 3), even if with some contrasting evidences; ii) the role of an additional gene (probably different from *ASIP*). Investigating these hypotheses, the goat could become an interesting model for the study of the mechanisms regulating melanin synthesis in mammals.

## Methods

### Animals

Blood, hair or milk samples were collected from 153 Girgentana (from five Sicilian farms), 50 Maltese (40 from five Sicilian farm and 10 from one Sardinian farm), 39 Derivata di Siria (from three Sicilian farms), 28 Murciano-Granadina (from one farm in the Granada province, Spain; 15 with solid *caoba *and 13 with solid black coat colour), 29 Camosciata delle Alpi (from three farms in the North of Italy) and 18 Saanen (from three farms in the North of Italy) goats. Pictures or coat colour descriptions were available for all animals except for 51 Girgentana goats that were excluded from the association analysis between coat colour and *MC1R *genotypes. The 10 Maltese animals obtained from the Sardinian farm were from a flock, in which Derivata di Siria blood was probably introduced in the past. In this flock we identified a goat with a Maltese-like pattern but that had reddish-head instead of black cheeks and ears (Figure 5). This goat was not listed among the 50 analysed Maltese animals because was not registered as Maltese due to its out-of-type colour. A cross between a Maltese buck (black; included in the list of 50 Maltese animals) selected in this flock and this red goat gave birth to three other goats, two black and one red (the same as the mother; Figure 5).

### Sequencing of the *MC1R *gene

DNA was extracted using a standard phenol-chloroform protocol for blood [45], the Wizard^® ^Genomic DNA Purification kit for blood and milk (Promega Corporation, Madison, WI) or rapid extraction methods for milk and hair roots [46]. Primers for caprine *MC1R *amplification and sequencing (Additional file 1) were designed with Primer 3 (Whitehead Institute for Biomedical Research, Cambridge, MA) from the published goat DNA sequence that only accomplish the coding region (GenBank accession number: Y13958) [47] and from the bovine *MC1R *complete gene sequence that includes 5'- and 3'-untranslated and flanking regions (GenBank accession number: AF445641) [9]. Sequences were obtained from 48 random goats across six breeds (Girgentana, 10; Maltese, 10; Derivata di Siria, 10; Murciano-Granadina, 6; Camosciata delle Alpi, 6; Saanen, 6). In addition, sequencing of the *MC1R *gene was carried out from the Maltese buck, the red Maltese-like goat and the three F_1 _animals obtained crossing these two goats. PCR was performed using a TGradient thermal cycler (Biometra, Goettingen, Germany) or a PT-100 thermal cycler (MJ Research, Watertown, MA, USA) in a volume of 20 μL containing 10–100 ng DNA template, 1 U DNA EuroTaq DNA polymerase (EuroClone Ltd., Paington, Devon, UK), 1× PCR Buffer, 2.5 mM dNTPs, 10 pmol of each primer and optimised MgCl_2_concentrations (from 2.0 to 2.5 mM). PCR profile was as follows: 5 min at 95°C; 35 amplification cycles of 30 s at 95°C, 30 s at 60/65°C, 30 s at 72°C; 5 min at 72°C. For the *MC1R *fragments sequencing 3–5 μL of PCR product was treated with 2 μL of ExoSAP-IT^® ^(USB Corporation, Cleveland, Ohio, USA) following the manufacturer's protocol. Cycle sequencing of the PCR products was obtained with the Big Dye v3.1 kit (Applied Biosystems, Foster City, CA, USA) and sequencing reactions, after a few purification steps using EDTA 0.125 M, Ethanol 100% and Ethanol 70%, were loaded on an ABI3100 Avant sequencer (Applied Biosystem). All sequences were visually inspected, edited, assembled, and aligned with the help of the BioEdit software v. 7.0.5.2 http://www.mbio.ncsu.edu/BioEdit/bioedit.html and the CodonCode Aligner software http://www.codoncode.com/aligner.

### SNP genotyping

To analyse the five point mutations found by sequencing, four different PCR-RFLP methods were established using primer pairs 2-ch7, E1-2, and A-2 and four different restriction endonucleases (Additional file 1). PCR was performed as described above and in Additional file 1. Both c.183C>T and c.242C>T SNPs disrupt/create a GGCC restriction site for *Hae*III endonuclease; therefore, we could genotype our samples for both mutations, using the same 2-ch7 primer pair, amplifying a 169 bp fragment and performing the same PCR-RFLP reaction. The nonsense mutation (c.673C>T) was analysed using primer pair E1-2, that amplifies a 267 bp fragment, and *Xba*I restriction enzyme (TCTAGA). The c.748T>G mutation was analysed amplifying a fragment of 123 bp, using primer pair A-2 with a forward primer that creates an artificial restriction site (ACGT) for *Tai*I endonuclease. The same PCR product was subjected to a further PCR-RFLP reaction with *Hae*III to investigate the c.801C>G point mutation. Additional file 2 reports the electrophoretic patterns of the investigated mutations.

### Sequence analysis and statistics

*In silico *functional analysis of missense mutations was obtained using PANTHER [31] whose predictions have been experimentally validated [48]. PANTHER estimates the likelihood of a particular non-synonymous (amino-acid changing) coding SNP to cause a functional impact on the protein. It calculates the substitution position-specific evolutionary conservation (subPSEC) score based on an alignment of evolutionarily related proteins [31-33]. The probability that a given variant will cause a deleterious effect on protein function is estimated by P_deleterious_, such that a subPSEC score of -3 corresponds to a P_deleterious _of 0.5 [48]. The subPSEC score is the negative logarithm of the probability ratio of the wild-type and mutant amino acids at a particular position. PANTHER subPSEC scores are continuous values from 0 (neutral) to about -10 (most likely to be deleterious). For the analysed animals, haplotypes including the five SNPs within the goat *MC1R *gene were inferred using the PHASE program v. 2.1 [49].

A median-joining network [50] for these haplotypes was constructed using Network v. 4.510 [51], including the sheep *MC1R *coding sequence (GenBank accession number: Y13965) [12]. When appropriate, association between SNPs and coat colours was tested using 2 × 2 contingency tables with Fisher exact test implemented in the procedure FREQ of SAS version 8.02 (SAS Institute Inc. Cary, NC, USA).

## Authors' contributions

LF conceived and took part in designing the study, analysed the sequences, contributed to the sampling, coordinated and organized the laboratory work, drafted the manuscript and partially funded the study. FB extracted the DNA, sequenced, read the sequences, genotyped the SNPs, prepared tables, figures and data and contributed to draft the manuscript. VRi contributed to the sampling and photographic documentation of the Sicilian breeds and data. SD sampled Camosciata delle Alpi and Saanen breeds and prepared photographic documentation and data. EGG sampled Murciano-Granadina breed and contributed to the SNP genotyping and sequencing. RF contributed to the sampling of Sicilian breeds and was involved in the design of the study. VRi, SD, RF, RD and VRu revised critically the manuscript and data. VRu supervised the work and was involved in the design of the study. BP sampled the Sicilian breeds, prepared photographic documentation, supervised, took part in designing the work and funded the study. All authors reviewed the manuscript and accepted the final version.

## Supplementary Material

Additional file 1 Primer sequences, PCR conditions for *MC1R *sequencing and PCR-RFLP analyses Click here for file

Additional file 2 Electrophoretic patterns of the goat *MC1R *PCR-RFLP analyses Click here for file
