# Supplementary material for: Missense and nonsense mutations in melanocortin 1 receptor (MC1R) gene of different goat breeds: association with red and black coat colour phenotypes but with unexpected evidences
Source: BMC Genet. 2009 Aug 25;10:47. doi: 10.1186/1471-2156-10-47 (PMC2748843; doi:10.1186/1471-2156-10-47)
Supplement: Additional file 1 — Primer sequences, PCR conditions for MC1R sequencing and PCR-RFLP analyses [file 1471-2156-10-47-S1.pdf]

Additional file 1 - Primer sequences, PCR conditions for *MC1R* sequencing and PCR-RFLP analyses

Primer pair	Forward primer and *reverse primer* (5'-3')	Fragment length (bp)	PCR conditions[1]	Use
ch5	aaacgatggtcagggagtg<br>*accgcactgtggcttctc*	597	63/2.5/TG	Sequencing
ch7	aagatgccaaggaaggctc<br>*ctgacgctcaccagcaagt*	592	63/2.0/TG	Sequencing
2-ch7	agtgcctggaggtgtccatcc<br>*ctgacgctcaccagcaagt*	169	62/2.0/TG	PCR-RFLP analysis with *Hae*III (c.183C>T and c.242C>T)
ch9	agccatgagttgagcaggac<br>*caggacaccagcctccag*	376	62/1.5/TG	Sequencing
2-4K	agtgcctggaggtgtccatcc<br>*gcctgggtggccaggaca*	229	62/2.0/PT	Sequencing
ch10	gtgagcgtcagcaacgtg<br>*acatagaggacggccatcag*	365	59/2.0/TG	Sequencing
4K-E1	caagaaccgcaacctgcact<br>*cagcaggatggtgagggtgg*	550	65/2.0/PT	Sequencing
E1-2	ctcgttggcctcttcatagc<br>*gaagttcttgaagatgcagcc*	267	62/2.0/PT	Sequencing; PCR-RFLP analysis with *Xba*I (c.673C>T)
E1-3	ctcgttggcctcttcatagc<br>*tgctggtcatgaagtccttg*	685	60/2.0/PT	Sequencing
A-2	gctgccaccctcaccatcctgctgggcgac<br>*gaagttcttgaagatgcagcc*	123	64/2.0/PT	PCR-RFLP analysis with *Tai*I (c.748T>G) and *Hae*III (c.801C>G)
ch8	accccctcatctatgccttc<br>*aaagatgggaaggaggttgc*	586	65/2.0/PT	Sequencing

[1] PCR conditions describe annealing temperature/$MgCl_2$ concentration (mM)/Thermal cycler (TG= TGradient thermal cycler - Biometra, Göttingen, Germany; PT= PT-100 thermal cycler - MJ Research, Watertown, MA, USA).
