# Supplementary material for: Missense and nonsense mutations in melanocortin 1 receptor (MC1R) gene of different goat breeds: association with red and black coat colour phenotypes but with unexpected evidences
Source: BMC Genet. 2009 Aug 25;10:47. doi: 10.1186/1471-2156-10-47 (PMC2748843; doi:10.1186/1471-2156-10-47)
Supplement: Additional file 2 — Electrophoretic patterns of the goat MC1R PCR-RFLP analyses [file 1471-2156-10-47-S2.pdf]

Additional file 2 – Electrophoretic patterns of the goat *MC1R* PCR-RFLP analyses

c.183C>T and c.242C>T
***Hae*III**

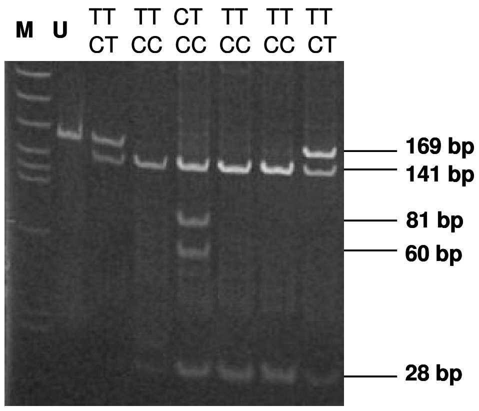

c.673C>T
***Xba*I**

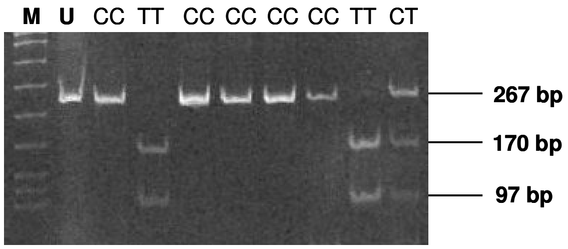

c.748T>G
***Tai*I**

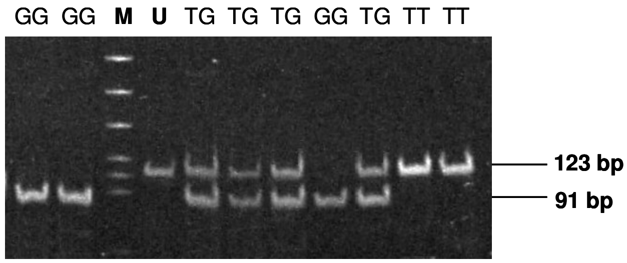

c.801C>G
***Hae*III**

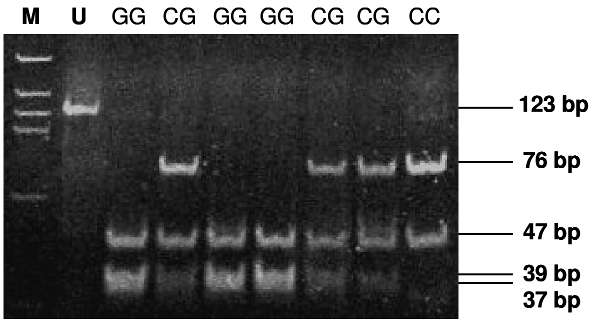

M= molecular weight marker VIII (Roche Diagnostic), U= undigested products, genotypes are indicated above each lane.
